# Lactate-mediated medium-chain fatty acid production from expired dairy and beverage waste

**DOI:** 10.1016/j.ese.2024.100424

**Published:** 2024-04-23

**Authors:** Bin Bian, Wenxiang Zhang, Najiaowa Yu, Wei Yang, Jiajie Xu, Bruce E. Logan, Pascal E. Saikaly

**Affiliations:** a Water Desalination and Reuse Center (WDRC), King Abdullah University of Science and Technology (KAUST), Thuwal 23955-6900, Kingdom of Saudi Arabia; b Environmental Science and Engineering Program, Biological and Environmental Science and Engineering (BESE) Division, King Abdullah University of Science and Technology (KAUST), Thuwal 23955-6900, Kingdom of Saudi Arabia; c Department of Civil and Environmental Engineering, The Pennsylvania State University, University Park, PA, 16802, USA; d Research Centre of Ecology & Environment for Coastal Area and Deep Sea, Southern Marine Science and Engineering Guangdong Laboratory (Guangzhou), Guangzhou, 511458, China; e School of Marine Science, Ningbo University, Ningbo, 315211, China

**Keywords:** Microbial chain elongation, Medium chain carboxylic acids, Food waste, Fermentation

## Abstract

Fruits, vegetables, and dairy products are typically the primary sources of household food waste. Currently, anaerobic digestion is the most used bioprocess for the treatment of food waste with concomitant generation of biogas. However, to achieve a circular carbon economy, the organics in food waste should be converted to new chemicals with higher value than energy. Here we demonstrate the feasibility of medium-chain carboxylic acid (MCCA) production from expired dairy and beverage waste via a chain elongation platform mediated by lactate. In a two-stage fermentation process, the first stage with optimized operational conditions, including varying temperatures and organic loading rates, transformed expired dairy and beverage waste into lactate at a concentration higher than 900 mM C at 43 °C. This lactate was then used to produce >500 mM C caproate and >300 mM C butyrate via microbial chain elongation. Predominantly, lactate-producing microbes such as *Lactobacillus* and *Lacticaseibacillus* were regulated by temperature and could be highly enriched under mesophilic conditions in the first-stage reactor. In the second-stage chain elongation reactor, the dominating microbes were primarily from the genera *Megasphaera* and *Caproiciproducens*, shaped by varying feed and inoculum sources. Co-occurrence network analysis revealed positive correlations among species from the genera *Caproiciproducens*, *Ruminococcus*, and *CAG*-352, as well as *Megasphaera*, *Bacteroides*, and *Solobacterium*, indicating strong microbial interactions that enhance caproate production. These findings suggest that producing MCCAs from expired dairy and beverage waste via lactate-mediated chain elongation is a viable method for sustainable waste management and could serve as a chemical production platform in the context of building a circular bioeconomy.

## Highlights

• Dairy/beverage waste was converted into butyrate and caproate via chain elongation.
• Lactate-producing microbes were regulated and enriched via temperature tuning.
• 85% caproate selectivity was achieved with lactate as the sole electron donor.
• Dominant chain elongators were regulated by different substrates and inocula.

## 1 Introduction

According to the Food and Agriculture Organization of the United Nations, one-third of the world's annual food production, totaling 1.3 billion tons, is either wasted or landfilled without generating any value [1]. Fruits/vegetables (22%) and dairy products (19%) usually represent the most common food waste generated from retail, food service, and households [2]. Commonly, food waste is rich in organic matter and could generate environmental contaminants, such as leachate and odors, without proper treatment [3]. To recover resources/values and avoid environmental contaminations, anaerobic digestion (AD) is utilized for the treatment of food waste, generating biogas for heating and electricity [4]. However, rapid acidification and slow hydrolysis can usually lead to the low efficiency of AD for the treatment of high-strength organic wastes, resulting in lower methanogenic activities and methane production [5]. Thus, novel bioprocesses are needed for resource recovery from high-strength organic waste streams, such as dairy/beverage waste [6], to promote a circular economy.

In recent years, a novel fermentation platform called microbial chain elongation has been developed to harness the potential of certain microbes to produce medium-chain carboxylic acids (MCCAs, C6–C10) from high-strength organic wastes [7]. MCCAs commonly have higher market prices (e.g., caproic acid > $1.6 kg^−1^) [8] compared to biogas and could be utilized for various industrial and agricultural applications, such as sustainable antimicrobials [9], precursors for liquid biofuel production [10], and feed additives for livestock growth [11]. So far, the feasibility of a chain elongation platform has been demonstrated for the valorization of various organic waste feedstocks, including but not limited to thin stillage [12], liquor/beer brewing wastewater [13,14], acid whey [15], and cheese whey [16,17], through one- or two-stage anaerobic fermentation processes mainly driven by ethanol or lactate as intermediates. Considering the naturally enriched lactate-producing bacteria in dairy products [18] and the easier conversion of carbohydrate-rich organic wastes to lactate compared to ethanol [15,19], lactate-driven chain elongation has been chosen for the valorization of some dairy wastes [15,16].

So far, the most common dairy waste treated via lactate-driven chain elongation is acid whey [15] and cheese whey [16,17]. Different pH [20], hydraulic retention times [17], MCCA extraction methods [15], and single-stage or two-stage processes [15] have been explored with lactate-driven microbial chain elongation. Two-stage lactate-driven chain elongation has been demonstrated to generate higher MCCA concentration from acid whey than single-stage processes [15]. In a two-stage lactate-driven chain elongation study, acid whey waste was utilized as the feedstock for the first-stage fermentation reactor at 50 °C to generate a high concentration of lactate for the efficient MCCA production in the second stage with chain elongation microbiomes enriched using ethanol-rich fermentation beer [15]. However, the systematic optimization of the two-stage lactate-driven chain elongation, especially operation parameters for lactate production in the first stage and the impacts of feedstock and inocula in shaping the microbial community and MCCA production in the second stage, has seldom been studied. Considering the significantly higher concentrations of fat and proteins (∼3.5%, of which 80% are casein proteins and 20% whey proteins [6]) in expired milk/yogurt/beverage waste and the associated higher treatment challenges compared to acid/cheese whey wastes, it is thus essential to develop a two-stage chain elongation platform, allowing the optimization of operation conditions separately in each stage to achieve satisfactory MCCA production.

To achieve this objective, a two-stage chain elongation system was developed for the MCCA production from complex expired milk/yogurt/beverage waste. We systematically explored the impacts of operation parameters on lactate production in the first-stage fermentation reactor, as well as the influences of substrates and inocula on the second-stage chain elongation. The temperature and organic loading rate were first optimized to enhance lactate concentration and production rate in the fermentation reactor, the effluent from which was then used as a feedstock for the chain elongation reactor and compared with different substrates to examine their impact on shaping the microbial communities.

## 2 Materials and methods

### 2.1 Fermentation reactor construction and operation

A fermentation reactor (5 L working volume) with a height of 28 cm and internal diameter of 19.5 cm was established and inoculated with 200 mL anaerobic sludge from a continuous stirred-tank reactor (CSTR) as previously reported [21]. Expired milk, yogurt, and fruit (mainly orange) juice (total level of chemical oxygen demand (COD) > 200 g L^−1^) received from a local food company were mixed at a ratio of 5:3.2:1.8 based on the actual percentage of each expired food in the total waste and then fed without any pretreatment to the fermentation reactor that was operated in batch mode for initial acclimatization. After one month of batch operation, the fermentation reactor was operated in continuous mode without biomass recirculation or settling and fed with the waste mixture that was pretreated at 100 °C for 20 min to remove the excessive protein content through centrifugation at 4000×*g* for 10 min (Scheme). The centrifuged medium (CM, total COD level 50–80 g L^−1^) was then stored at 4 °C and used as a feedstock for the fermentation reactor.

**Scheme:**
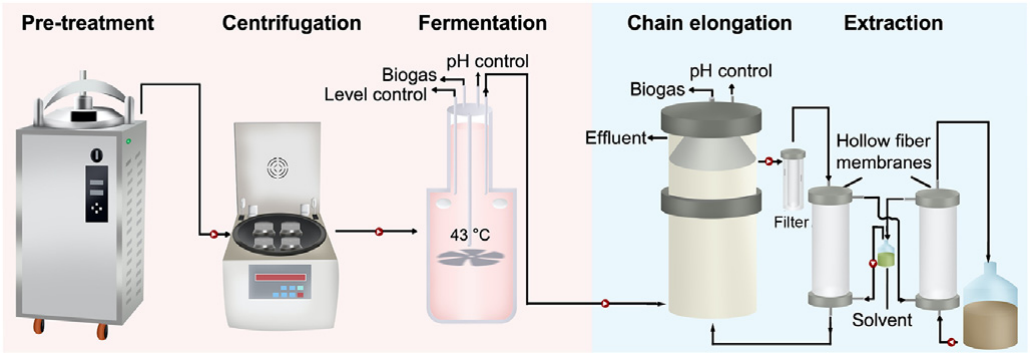
Schematic of lactate-mediated MCCA production involving two-stage fermentation and chain elongation processes.

Continuous operation of the first-stage fermentation reactor was divided into five periods (F–I to F–V) to optimize operation conditions (i.e., temperature and organic loading rate) for enhancing lactate concentration and production rate. The optimization details, including increasing the operation temperature (from 30 to 35 and then 43 °C, F–I to F-III) and the organic loading rate by adjusting the hydraulic retention time (10.13039/100009982HRT, F-10.13039/501100000026IV) and CM dilution ratio (F–V), could be found in Table 1 and Supporting Information (SI). The pH in the fermentation reactor was maintained at 5.2 by periodically adding sodium hydroxide solution (1 M), and the agitation was fixed at 100 rpm by a control panel (Huisen Bio, China).

**Table 1:** Experimental approach and operating conditions for the fermentation and chain elongation reactors.

Operation parameters for the fermentation reactor					
Periods	Days	Temperature (°C)	HRT (d)	Medium dilution (CM:TW)^a^	Organic loading rate (g COD L^−1^ d^−1^)
F–I	0–38	30	4	1:2	4.9
F-II	39–54	35	4	1:2	5.1
F-III	55–86	43	4	1:2	4.2
F-IV	87–134	43	2	1:2	8.4
F–V	135–166	43	2	CM without dilution	28.1
Operation parameters for the chain elongation reactor					
Periods	Days	Temperature (°C)	HRT (d)	Medium (mM C)^b^	Organic loading rate (g COD L^−1^ d^−1^)
CE-I	0–14	35	2.5	EtOH 800, Ac 400	25.7
CE-II	15–28	35	2.5	EtOH 800, Ac 400, LA 300	29.4
CE-III	29–42	35	2.5	EtOH 400, Ac 200, LA 600	25.7
CE-IV	43–56	35	2.5	EtOH 400, LA 1050	28.0
CE-V	57–70	35	2.5	LA 1050	18.2
CE-VI	71–105	35	2.5	Effluent from fermentation reactor containing LA 810–840	19.2

a CM: centrifuged medium, TW: tap water.

b EtOH: ethanol; Ac: acetate; LA: lactic acid.

### 2.2 Chain elongation reactor construction and operation

The chain elongation reactor was scaled up to a working volume of 10 L from an up-flow reactor (2.25 L), with an internal diameter of 11 cm and a height of 126 cm. The ethanol/acetate mixture was used as the initial feed substrates and electron donors for the enrichment of chain elongation microbiome and MCCA production based on our previous experience [7,15]. To examine the impacts of different feed substrates on MCCA production, microbial community evolution, and the feasibility of a two-stage system for MCCA from expired dairy/beverage waste, the operation of the chain elongation reactor was divided into six periods (CE-I to CE-VI, Table 1), by gradually switching the feed substrate from ethanol/acetate to lactate only, and then to the effluent from the fermentation reactor above. The details of feed substrates during Periods CE-I to CE-VI can be found in Fig. 2a, Table 1, and Supplementary Information (SI). The chain elongation reactor was initially inoculated in Period CE-I with a mixture of activated sludge from the municipal Wastewater Treatment Plant at King Abdullah University of Science and Technology, anaerobic sludge from a lab-scale anaerobic membrane bioreactor, and sediments from a lab-scale fermentation reactor. An additional source of inoculum using local sheep rumen was added between Periods CE-III and CE-IV to examine the impact of inocula on the MCCA production and microbial community evolution in the second-stage chain elongation reactor. Plastic biocarriers were added to the chain elongation reactor to improve biomass retention. The HRT for the chain elongation reactor was set at 2.5 days, and 1–3 g L^−1^ yeast extract was added during the whole experiment except in Period CE-VI based on previous studies [22,23]. The temperature was maintained at 35 °C using a recirculating water bath (MP-5H, Hinotek, China). The broth pH was maintained at 5.5–6.0 by an automatic pH controller (400 pH/ORP, Cole-Parmar, the United States) and a dosing pump to add sodium hydroxide solution (1 M) at the top of the reactor. The biogas was collected and recorded by a flow gas meter (TG05, Ritter, Germany). An external extraction unit made of hollow-fiber membranes was constructed for the continuous forward and reverse extraction of MCCAs from the chain elongation bioreactor (See SI), similar to our previous report [7].

### 2.3 Microbial community analysis

Biomass samples were collected from the fermentation reactor before switching to continuous-flow operation and at the end of each period of continuous-flow operation. Similarly, biomass samples of the original inoculum, rumen inoculum, and at the end of each period (except CE-II and CE-III) were collected for the chain elongation reactor. The variable region 4–8 (abeV48-A) of the archaea/bacteria/eukarya 16S/18S rRNA gene was sequenced using a custom protocol. The detailed procedure for DNA extraction, sequencing library preparation, sequencing, and processing of sequence reads were provided in the SI. Co-occurrence network analysis was conducted for the microbial communities collected from the fermentation and chain elongation reactor during Periods F–I to F-VI and CE-I to CE-VI, respectively. Multiple testing correction was conducted via false discovery rate (FDR) estimation using “igraph” and “psych” packages in RStudio [24]. The correlations between operational taxonomic units (OTUs) were considered significant when Spearman's correlation coefficient *r* > 0.6 and *p* < 0.01. The fast-greedy modularity optimization was applied to calculate the modular structure of the phylogenetic molecular ecological networks of microbial communities in the fermentation and chain elongation reactors [25].

### 2.4 Liquid and gas sample analysis

Liquid samples were collected every two days from the fermentation reactor and twice a week from the chain elongation reactor and filtered using 0.22 μm syringe filters (VWR). High-performance liquid chromatography (HPLC, Waters) [26] was used for the detection of volatile fatty acids (VFAs) and lactate in the filtrate. Since the exact component composition in the fermentation-influent mixture (i.e., expired milk/yogurt/juice) is not known, the average selectivity of each product in the fermentation effluent was calculated by dividing the concentration of the formed product by the total product concentration (based on mM C) [14], while in the chain elongation reactor it was calculated by dividing the concentration of electrons in the formed products (butyrate and caproate) by the net consumed electrons from lactate [27,28]. Gas chromatograph-mass spectrometry (GC-MS, 7890A, Agilent Technologies) equipped with a flame ionization detector (FID) [29,30] was used for the detection of ethanol in the filtrate. Gas samples were periodically analyzed using gas chromatography (GC, model 310C, SRI Instruments) [31].

## 3 Results and discussion

### 3.1 Effect of temperature on the performance of the fermentation reactor

**Fig. 1:**
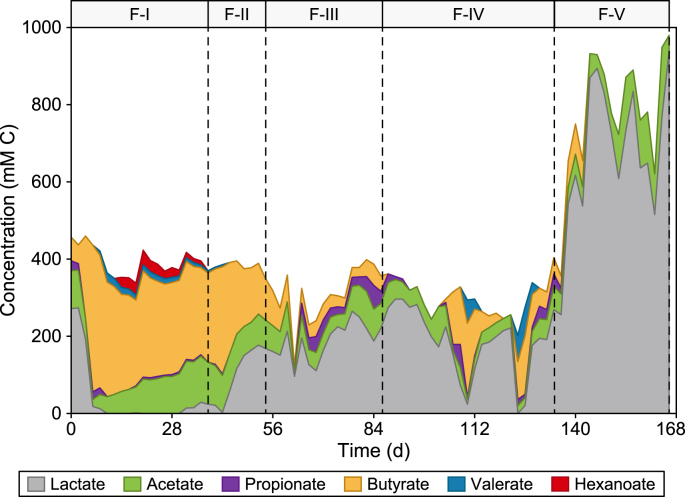
Stacked area charts for broth concentration of lactate and volatile fatty acids during Periods F–I to F–V in the fermentation reactor. F–I: 30 °C, HRT 4 d, CM:TW 1:2; F-II: 35 °C, HRT 4 d, CM:TW 1:2; F-III: 43 °C, HRT 4 d, CM:TW 1:2; F-IV: 43 °C, HRT 2 d, CM:TW 1:2; F–V: 43 °C, HRT 2 d, CM without dilution.

The temperature had a notable effect on the product spectrum in the fermentation reactor (Fig. 1), switching the main component from butyrate at 30 °C (Period F–I) to lactate at 43 °C (Period F-III). The maximum concentration of butyrate (380.4 ± 10.8 mM C) at 30 °C (Period F–I) was achieved on day 6 with the diluted medium (CM:tap water (TW) = 1:2), representing a production rate of 95.1 ± 2.7 mM C d^−1^ at an HRT of 4 d (Fig. 1; Fig. S1) and a butyrate selectivity of 87.2 ± 0.2% (based on mM C) within all lactate and VFAs in Period F–I (30 °C). However, the lactate production was severely suppressed (18.3 ± 0.3 mM C on day 6) at 30 °C with selectivity usually below 10% in the fermentation effluent (Fig. S2), which was reported to be insufficient for the efficient MCCA production through chain elongation [14]. Apart from lactate and butyrate, acetate (17.8 ± 0.5 mM C) and propionate (19.7 ± 3.4 mM C) were also measured. Considering 19.4 g COD L^−1^ in the influent, the maximum COD conversion efficiency to VFAs and lactate reached 88.0 ± 6.9% on day 6 at 30 °C (Period F–I, Fig. S2), which was slightly higher than a previous report using acid whey as the feedstock [15].

After switching the operation temperature to 35 °C (Period F-II) and then 43 °C (Period F-III), the product spectrum was dramatically shaped, as the average butyrate concentration after stabilization dropped from 263.9 ± 44.1 mM C at 30 °C to as low as 38.6 ± 14.8 mM C at 43 °C (*t*-test, *p* value < 0.01). On the contrary, the lactate concentration significantly increased from almost 0 mM C to an average of 187.9 ± 49.0 mM C (*t*-test, *p* value < 0.0001), with the highest concentration reaching 264.9 ± 6.3 mM C on day 78 in Period F-III (Fig. 1). The average lactate concentration could be maintained at 187.6–264.9 mM C after stabilization at 43 °C (Period F-III), which was reported to be sufficient to initiate the chain elongation process [14,22]. 50.7 ± 1.2% of the influent COD was converted to lactate on day 78, and the highest lactate selectivity (73.8 ± 3.6% based on mM C) in the effluent was obtained on day 74 at 43 °C. The average lactate selectivity at 43 °C (63.8 ± 9.7%, Period F-III) was significantly higher compared to 30 °C (0.7 ± 1.5%, Period F–I, Fig. S2) (*t*-test, *p* value < 0.01), but was slightly lower than that reported by Xu et al. using acid whey for the lactate fermentation at 50 °C [15] and close to what was achieved by Bühlmann et al. [32] with food waste mix at 40–50 °C. The improved lactate production and selectivity at 43 °C during Period F-III indicated the potential change in the fermentation pathways. Homolactic fermentation was believed to play a major role in lactate production at 43 °C during Period F-III with the enrichment of homolactic microorganisms, such as *Lactobacillus* and *Lacticaseibacillus* (section 3.4.1) [33]. However, apart from the main product lactate, 12.0–96.2 mM C of acetate and 0–61.1 mM C of propionate were also observed in the effluent at 43 °C (Period F-III), indicating the potential involvement of heterolactic fermentation or lactate-consuming pathways involved at 43 °C [33]. The heterolactic fermentation or lactate-consuming pathways was partially demonstrated by the higher production of H_2_ and CO_2_ as byproducts with butyrate and acetate during Period F–I (1.3 L CO_2_ d^−1^ and 1 L H_2_ d^−1^) and F-II (0.9 L CO_2_ d^−1^ and 0.8 L H_2_ d^−1^), compared to 0.4 L CO_2_ d^−1^ and 0.2 L H_2_ d^−1^ during Period F-III in the gas phase [33]. The maximum percentage (74.3 ± 2.6% to 79.6 ± 2.6%) of COD (16.7 g COD L^−1^ in the influent) converted to VFAs and lactate occurred between day 78 and day 84 and was slightly lower than what was obtained at 30 °C (Period F–I), which was mainly due to the lower COD conversion factor of 1 g lactic acid (1.066 g COD) compared to 1 g butyric acid (1.816 g COD). Increasing the operation temperature for the fermentation reactor in this study improved the lactate production/selectivity and potentially enhanced MCCA production in the following chain elongation reactor.

### 3.2 Effect of increasing the organic loading rate on the performance of the fermentation reactor

Increasing the organic loading rate to further enhance the lactate production was first demonstrated by reducing the HRT from 4 days during Period F-III to 2 days during Period F-IV (both at 43 °C and medium dilution CM:TW = 1:2). The lactate concentration immediately increased from 220.5 ± 1.6 to 274.4–296.3 mM C (days 88–92) after reducing the HRT. The highest lactate concentration of 296.3 ± 7.6 mM C was obtained on day 90 in Period F-IV (HRT = 2 d) (Fig. 1), representing a 35% increase compared to Period F-III (HRT = 4 d). Similarly, the production rate of lactate increased by 124%, from 66 ± 2 mM C d^−1^ on day 78 in Period F-III (HRT = 4 d) to 148 ± 4 mM C d^−1^ on day 90 in Period F-IV (HRT = 2 d) (Fig. S1). The highest lactate selectivity reached 86.2 ± 0.1% on day 94 and 87.2 ± 0.2% on day 120 before the performance of the fermentation reactor deteriorated between days 108–112 and 124–126 due to a dysfunction in the pH probe (pH > 10) and the activity of lactate-producing microbes was significantly suppressed. However, the lactate concentration recovered, reaching more than 200 mM C with a selectivity of more than 70% within ten days after adjusting the pH back to 5.0–5.5 (Fig. 1). After lowering the HRT, the overall COD conversion efficiency slightly declined to reach 68.2 ± 1.8% on day 90, which could be due to the washout of more biomass from the fermentation reactor at the start of Period F-IV (HRT = 2 d). However, the COD conversion efficiency gradually recovered to 80.4 ± 1.1% on day 134, and lactate accounted for 51.5 ± 0.2% of the total COD conversion in Period F-IV (HRT = 2 d). The hydrogen and CO_2_ gas production during Period F-IV remained low (0.7 L CO_2_ d^−1^ and 0.4 L H_2_ d^−1^) due to homolactic fermentation [33]. These results suggest that the stability of the fermentation reactors was not impacted significantly by increasing the organic loading rate by lowering the HRT.

To further improve the lactate production in the fermentation reactor, the organic loading was further increased using non-diluted feedstock in Period F–V (days 135–166) [34]. Lactate concentration immediately increased from 255.9 ± 10.8 mM C on day 136–544.0 ± 6.6 mM C on day 138. It continued to increase, reaching a maximum value of 937.8 ± 18.9 mM C on day 166 in Period F–V (no dilution) (Fig. 1), around 3.7-fold higher than day 136. The higher lactate concentration using non-diluted CM feedstock in this study was close to what was achieved by Xu et al. using acid whey as feedstock [15] and could trigger higher MCCA production in the second-stage chain elongation process [14]. Apart from lactate concentration, the selectivity of lactate in the product profile was also improved in Period F–V, as only lactate and acetate were detected after day 142. The average lactate selectivity reached 87.6 ± 5.9% during Period F–V (Fig. S2), reaching 96.2 ± 0.3% on day 146, and was significantly higher compared to that during Period F-IV (*t*-test, *p* value < 0.05). This lactate selectivity was comparable to or even higher than what has been reported with other studies on food waste fermentation [18,32], which was beneficial for the subsequent MCCA production in the chain elongation reactor. However, the average conversion efficiency of influent COD to VFAs and lactate was considerably lower at 47.9 ± 6.2% in Period F–V. This might be attributed to the significant COD loss for the gas production (1.7 L CO_2_ d^−1^ and 0.9 L H_2_ d^−1^) and the higher amounts of solid residues in the fermentation effluent during Period F–V. Overall, the lactate selectivity and production from expired dairy/beverage waste could be significantly enhanced by increasing the temperature to 43 °C and the organic loading rate, which represented a feasible strategy to provide feed source and electron donors for higher MCCA production in the following chain elongation reactor.

### 3.3 Performance of chain elongation reactor with different medium composition as feedstock

**Fig. 2:**
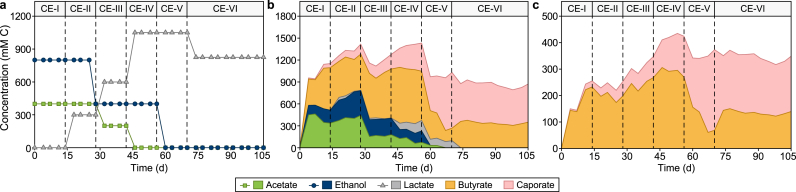
**a**, Line chart for the feeding medium composition into the chain elongation reactor during CE-I to CE-VI. The feed medium for Period CE-VI was collected from the first-stage fermentation reactor (around 100 L), and its actual concentration was measured before being fed into the chain elongation reactor. **b**, Stacked area chart for broth concentration of ethanol, volatile fatty acids, and lactate in the chain elongation reactor during Periods CE-I to CE-VI. **c**, Stacked area chart for the production rate of butyrate and caproate during Periods CE-I to CE-VI in the chain elongation reactor, operated at a fixed HRT of 2.5 days.

To identify the impacts of different feed sources and electron donors on MCCA production in the chain elongation reactor, chain elongation microbes enriched from the mixed sludge sources were first fed with a synthetic medium containing 800 mM C ethanol (EtOH) and 400 mM C acetate (Ac) in Period CE-I (Fig. 2a). Lactic acid (LA) was added to the synthetic medium in Period CE-II and its concentration was gradually increased to reach a concentration of 1050 mM C in Period CE-V, while the concentrations of acetate and ethanol were gradually decreased to reach 0 mM C. Results showed that the concentration of acetate in the chain elongation effluent was 403.0 ± 47.6 mM C during Period CE-I (EtOH 800, Ac 400 mM C) and CE-II (EtOH 800, Ac 400, LA 300 mM C) (Fig. 2b), suggesting it was not consumed by microbes. Acetate likely had a negligible contribution to caproate production in the chain elongation reactor, as we only observed ethanol consumption during Period CE-I and ethanol/lactate during Period CE-II. Lactate, compared to ethanol, seemed to be favored by chain elongation microbes, as it was almost completely consumed in Period CE-II, while the consumption of ethanol significantly decreased from 646.3 ± 33.3 mM C during Period CE-I to 488.9 ± 47.0 mM C during Period CE-II (*t*-test, *p* value = 0.0016). Lactate played an important role in the caproate production despite a reduction of acetate and ethanol concentrations in the feed during the whole experiment period (Fig. 2a), as the average caproate concentration in the chain elongation effluent increased significantly from 105.3 ± 25.1 mM C in Period CE-II (EtOH 800, Ac 400, LA 300 mM C) to 613.2 ± 149.8 mM C in Period CE-V (LA 1050 mM C) (Fig. 2b) (*t*-test, *p* value = 0.0004). The highest butyrate concentration was observed on day 46 during Period CE-IV, reaching 764.8 ± 8.0 mM C using a synthetic medium containing 0 mM C acetate, 400 mM C ethanol, and 1050 mM C lactate. The corresponding caproate concentration was 261.6 ± 20.4 mM C on day 46 and increased to 761.4 ± 30.0 mM C at the end of Period CE-V (LA 1050 mM C). The concentrations of butyrate and caproate in the effluent of the chain elongation reactor during Period CE-V slightly exceeded the theoretical amount of lactate conversion to butyrate and caproate (700 mM C) through reverse *β*-oxidation, which could be attributed to the remaining ethanol/acetate from Period CE-IV (Fig. 2b) and the yeast extract that was added into the synthetic medium.

After gradual adaptation with a synthetic lactate-containing medium for ten weeks, the chain elongation reactor in Period CE-VI was only fed with unfiltered and unsterilized effluent generated from the fermentation reactor. The lactate concentration was measured at around 810–840 mM C in the fermentation reactor effluent (Fig. 2a), with a small amount of acetate (<50 mM C), solid residues, and non-digested COD. The lactate was completely consumed in the chain elongation reactor to mainly form caproate (524.5 ± 24.7 mM C) and butyrate (338.8 ± 20.9 mM C) (Fig. 2b), partially supporting our hypothesis above that lactate, compared to ethanol, seemed to be favored by chain elongation microbes in this study and the chain elongation reactor was not impacted by the complete removal of ethanol and acetate. The results from CE-V and CE-VI show that the caproate concentration and composition in the product profile was much higher with lactate as the sole substrate and electron donor than ethanol/acetate mixed feed in CE-I to CE-IV. The lactate-to-MCCA conversion ratio was higher than the theoretical value, possibly due to the microbial conversion of the remaining organics and residues in the fermentation reactor effluent. The biogas production (H_2_: ∼81%, N_2_: ∼3%, and CH_4_: 16%) from the chain elongation reactor also increased with the increase in the concentration of lactate, reaching 24–40 L d^−1^ in Periods CE-V and CE-VI (Fig. S3). The higher production of H_2_ than CH_4_ could be attributed to the acidic pH maintained in the reactor, which inhibits methanogens [35].

The maximum caproate concentration (14.8 g L^−1^, 764.8 ± 8.0 mM C) obtained with lactate as the sole electron donor in the chain elongation reactor in this study was comparable to or even higher than most studies on lactate-driven chain elongation process as shown in Table S1. The temperature-phased two-stage fermentation process for specific organic waste valorization might generate higher lactate concentration in the lactate-fermentation process [14,15,36], which triggers the higher MCCA production in the following chain elongation process. This could be attributed to the high relative abundance of lactate-producing microbes after enrichment in the fermentation reactor, avoiding the competition between lactate-, butyrate- and caproate-producing microbes (Section 3.4). The average caproate production rate in Period CE-VI reached 209.8 ± 9.9 mM C d^−1^ using the effluent from the fermentation reactor as feed for the chain elongation reactor (Fig. 2c), which was higher than what had been reported in similar studies using acid whey as the starting raw material [15,37,38].

The relatively high selectivity of caproate (∼85%) calculated by dividing the concentration of electrons in the formed caproate by the net consumed electrons from lactate in Period CE-VI was comparable to previous studies [15,[39], [40], [41]], leading to an 8.6-fold higher extraction rate for caproate (256 ± 15 vs. 30 ± 5 mmol C m^−2^ d^−1^ for butyrate) from the chain elongation reactor using a continuous membrane-liquid extraction unit [7,12]. This could further enhance the selectivity of longer-chain fatty acids after chain elongation, which could later reduce the cost of MCCA purification. Considering a relatively low membrane area-to-reactor volume of 0.025 m^2^ L^−1^, a low extraction efficiency of 3% was achieved in this study, which is expected to be further improved by increasing the membrane area-to-reactor volume to 0.5 m^2^ L^−1^ [7]. The results of this study demonstrated the feasibility of the two-stage lactate-driven chain elongation process for the successful valorization of expired dairy and beverage waste.

### 3.4 Microbial communities and co-occurrence analysis

#### 3.4.1 Dominance of lactate-producing genera in the fermentation reactor

The suspended biomass in the fermentation reactor before switching to continuous-flow operation was mostly dominated by the genus *Lactobacillus* (57.5%), followed by *Prevotella* 7 (14.6%) and *Lacticaseibacillus* (11.9%) (Fig. S4). *Lactobacillus* and *Lacticaseibacillus* are known for their capability of converting sugars and other organics into lactic acid [15,42], while *Prevotella* 7 have been reported to be correlated with lipid and carbohydrate metabolism to produce odd-chain fatty acid (OCFA) [43,44]. For the lactate-mediated chain elongation process, using dairy waste as the initial feed and inoculum source might be a good strategy for the fast enrichment of lactate-producing microbes in the fermentation reactor.

**Fig. 3:**
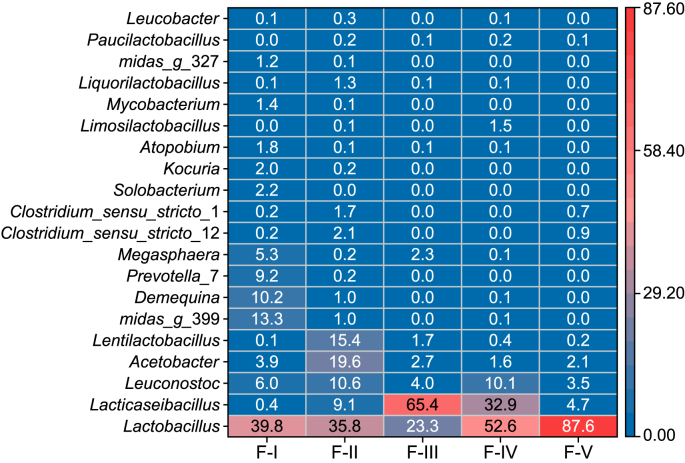
Heatmap of the relative abundance of the top 20 operational taxonomic units in the suspension of the fermentation reactor during Periods F–I to F–V. The taxa level shown on the left-hand side represents the genus level.

A heatmap of the top 20 OTUs in the suspended biomass of the fermentation reactor displayed the enrichment and dominance of lactate-producing microbes (*Lactobacillus*, *Lacticaseibacillus*, and *Leuconostoc* [45]) under different conditions (Fig. 3). The combined relative abundance of lactate-producing genera exhibited a significant improvement from 46.2% (30 °C, Period F–I) to 92.7% (43 °C, Period F-III), resulting in the generation of lactate as the major fermentation product at 43 °C (Fig. 1). In contrast, the relative abundance of OCFA producers (i.e., *Lentilactobacillus* [46] and *Prevotella* 7 [44]: 9.2–0%) and butyrate fermenters (*Megasphaera* [47]: 5.3–2.3%, and *Solobacterium* [48]: 2.2–0%) decreased with the temperature increase. The genus *Demequina*, capable of decomposing carbohydrates [49], also saw a reduction in its relative abundance from 10.2% to 0% with the temperature increase. Collectively, these results show that operating the fermentation reactor at a high temperature resulted in the dominance of lactate-producing microbes, which enhanced the overall conversion and utilization of carbohydrates into lactate (Fig. 1). This was demonstrated by the significantly higher average lactate concentration (187.9 ± 49.0 mM C) and selectivity (63.8 ± 9.7%) at 43 °C (Period F-III) compared to 30 °C (almost 0 mM C, 0.7 ± 1.5%, Period F–I) (*t*-test, *p* < 0.01) (Fig. 1; Fig. S2).

Increasing the organic loading rate further enhanced the dominance of lactate-producing microbes, with the combined relative abundance of *Lactobacillus*, *Lacticaseibacillus*, and *Leuconostoc* increasing from 92.7% (Period F-III, HRT of 4 days) to 95.6% (Period F-IV, HRT of 2 days) and 95.8% (Period F–V, CM with no dilution) (Fig. 3). The higher organic loading rate seems to favor the enrichment of *Lactobacillus* over *Lacticaseibacillus* as the relative abundance of *Lactobacillus* increased from 23.3% (Period F-III) to 52.6% (Period F-IV) and further to 87.6% (Period F–V), whereas the relative abundance of *Lacticaseibacillus* decreased from 65.4% (Period F-III) to 32.9% (Period F-IV) and finally reached 4.7% (Period F–V). The high dominance of *Lactobacillus* with increasing organic loading rate contributed to the increase in the percentage of lactate-C in the whole product composition, from 61.9% on day 86 in Period F-III to 66.6% on day 134 in Period F-IV and 95.7% on day 166 in Period F–V. The enrichment level of lactate-producing microbes in this study was higher than previous studies using food waste for MCCA production [19,50], but was comparable to a study with acid whey as feedstock in a two-stage chain elongation platform [15]. This demonstrates the advantage of using dairy waste to enrich lactate-producing microbes in a two-stage chain elongation platform, which allows the separate optimization of operation conditions for the first-stage fermentation reactor to achieve satisfactory lactate production for the subsequent MCCA production.

**Fig. 4:**
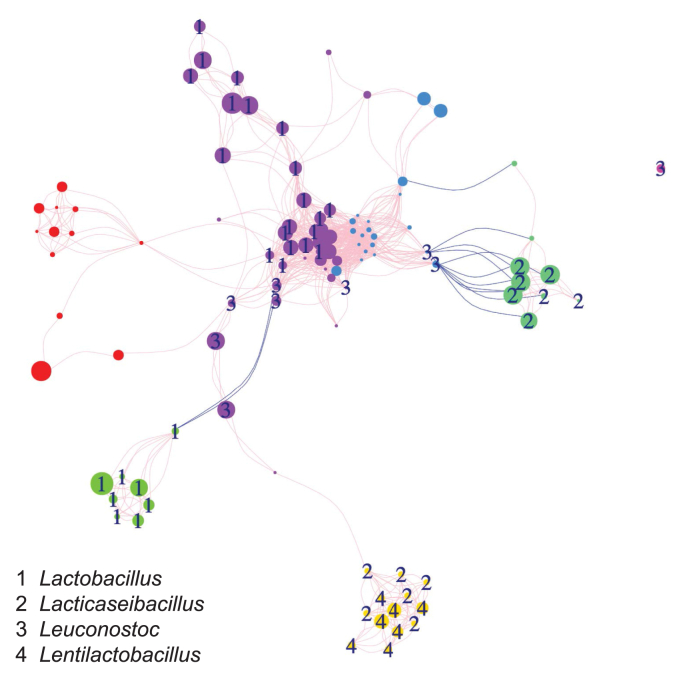
Co-occurrence networks between microbial OTUs in the fermentation reactor during Period F–I to F–V. Pink lines connecting nodes (with different sizes corresponding to their relative abundances) represent significantly positive correlations (*p* < 0.01) between microbes, while blue lines indicate significantly negative correlation coefficients between two different microbes. Nodes with the same color form a module in the network and represent different OTUs that are highly interconnected and have only a few connections outside the group.

The potential symbiotic interactions between the different microbial OTUs in the fermentation reactor were analyzed via a co-occurrence network (Fig. 4). A total of 833 strong correlations were discovered for 103 microbial species, among which 98% were positively linked. The majority of the species in the most dominant genera (i.e., *Lactobacillus* and *Lacticaseibacillus*) were found to have positive correlations within their genus for lactate production. However, species from the genus *Leuconostoc* were found to be mostly positively linked with species from the genus *Lactobacillus*, indicating their potential symbiotic interactions. Besides, over 30 positive correlations were observed between species within the genera clusters of butyrate-producer *Clostridium sensu stricto* [50] (red nodes), which explains the generation of butyrate in the fermentation reactor. However, further experiments are needed to confirm these interactions revealed by the co-occurrence network [51].

#### 3.4.2 Dominance of chain elongation microbes via lactate mediation

The initial anaerobic sludge inoculum used to seed the chain elongation reactor was mainly dominated by *Methanosarcina* (25.1%, Fig. S5). *Lactobacillus*, *Prevotella* 7, and *Lacticaseibacillus* became the dominant genera in Period CE-I (Fig. S6), mainly due to the introduction of sediments from the fermentation reactor. During this period, when ethanol and acetate (no lactate) were added to the reactor, the dominant chain elongator belonged to *Megasphaera*, but its relative abundance was relatively low (12.0%), which explains the low caproate production (Fig. 2b).

**Fig. 5:**
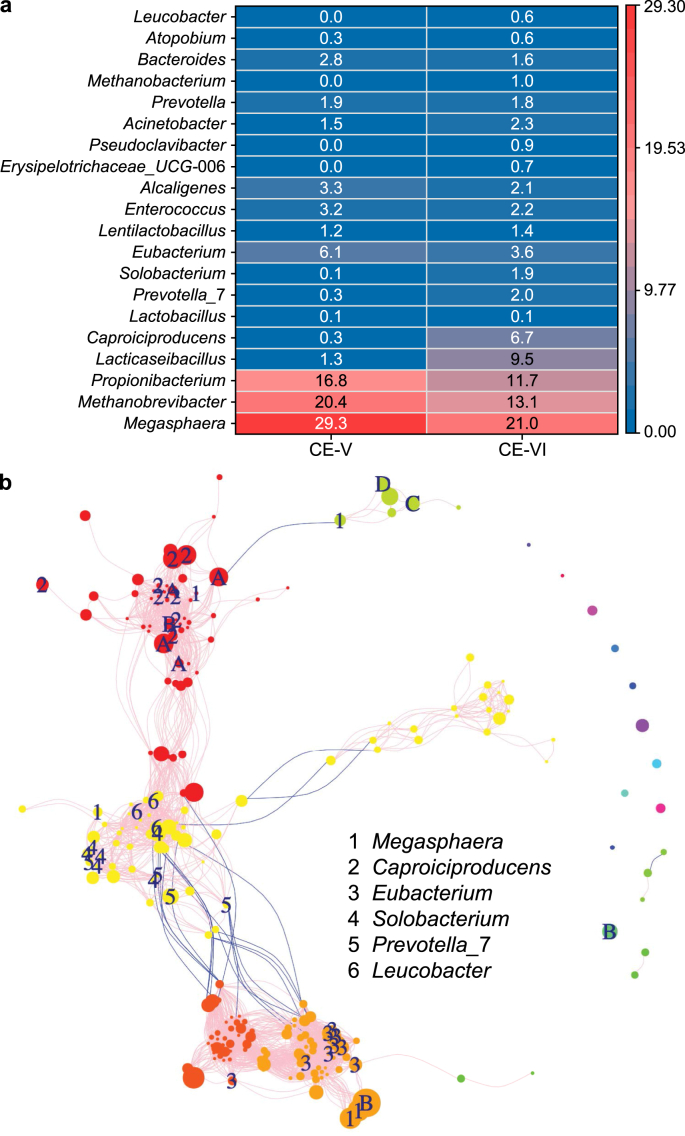
**a**, Heatmap of the relative abundance of the top 20 operational taxonomic units in the suspension of the chain elongation reactor operated with lactate only during Period CE-V and fermentation effluent in Period CE-VI. The taxa level shown on the left-hand side represents the genus level. **b**, Co-occurrence networks between microbial species in the chain elongation reactor during Periods CE-I to CE-VI. A, B, C, and D in the co-occurrence network represent the genera *Methanobacterium*, *Methanobrevibacter*, *Methanothrix*, and *Methanothermobacter*, respectively.

Efficient chain elongation did not start until lactate was utilized as the carbon source and sheep rumen was added as inoculum source between Period CE-II and CE-IV (Fig. 2b). The microbial communities in the sheep rumen were dominated by chain elongating bacteria (Fig. S5 and 24.2% *Megasphaera*, 2.7% *Eubacterium* [52], 2.6% *Alcaligenes* [53]), propionate producers (22.5% *Propionibacterium*) and methanogens (21.7% *Methobrevibacter*). After a series of adaption to lactate from Period CE-II (EtOH 800, Ac 400, LA 300 mM C) to CE-IV (EtOH 400, LA 1050 mM C) (Fig. 2a), the dominant chain elongating microbe shifted to *Caproiciproducens* (26.3%) [50] in the suspended biomass at the end of Period CE-IV (Fig. S6), which resulted in the increase of MCCA proportion, especially caproate (Fig. 2b), in the whole product spectrum. However, the operation of the microbial chain elongation reactor with lactate as the carbon source and mediator in Period V (LA 1050 mM C) shifted the dominance from *Caproiciproducens* to another chain-elongating bacteria, *Megasphaera* (29.3%, Fig. 5a), possibly because of the addition of sheep rumen, which had a high relative abundance of *Megasphaera* (24.2% *Megasphaera*, Fig. S5), and the complete elimination of ethanol. In a previous study, sheep or cow rumen was a common source for isolating *Megasphaera* spp. for MCCA production [54]. *Megasphaera hexanoica* has been reported to be capable of generating caproate from lactate as a pure culture in previous studies [55]. Further tests to feed the chain elongation reactor with the effluent from the fermenter, containing around 810–840 mM C lactate, did not change the dominance of chain elongating microbes (21.0% *Megasphaera*, 6.7% *Caproiciproducens*, and 3.6% *Eubacterium*) in Period CE-VI (fermentation effluent) compared to CE-V (LA 1050 mM C). The emergence of other chain elongators, such as *Acinetobacter* (2.3%) and *Alcaligenes* (2.1%) [53], was detected in Period CE-VI (fermentation effluent). These results suggest that the microbial community structure could be impacted and shaped by the change of substrates and inoculum sources, resulting in the dominance of chain elongators and higher production of MCCAs.

The interactions between different genera in the chain elongation reactor were illustrated by the co-occurrence network (Fig. 5b). A total of 239 species in the chain elongation reactor formed 2866 correlation connections between each other, among which 99% were positively linked with pink lines. The main chain elongation genera (*Megasphaera*, *Caproiciproducens*, and *Eubacterium*) and OCFA producers (*Prevotella* 7 and *Leucobacter*) were listed in the figure with node size proportional to their relative abundance. Over 85 positive correlations were found for the chain elongation species belonging to the genus *Caproiciproducens* within its own genus and with species from the genera *Ruminococcus* and *CAG-*352. Species belonging to the above three genera all belong to the family Ruminococcaceae, the correlations within which were not revealed in a previous study [36]. For the other caproic acid-producing bacteria, *Megasphaera* belongs to the family Veillonellaceae and was found to be positively correlated with species from the genera *Bacteroides*, whose members are commonly found in lactate-driven chain elongation microbiomes [56], and *Solobacterium*, a butyrate fermenter [48]. These tight interconnections indicated their potential symbiotic partnership, contributing to the final high concentration of MCCAs in Period CE-VI (fermentation effluent). Twenty-three negative correlations were found between the chain-elongating bacteria *Alcaligenes*, OCFA producer *Prevotella* 7, and lactate-producing species *Lacticaseibacillus*. Chain elongation species from the genus *Alcaligenes* may compete for lactate with *Prevotella* 7 and *Lacticaseibacillus* in the chain elongation reactor.

## 4 Conclusions and future directions

The feasibility and advantage of lactate-mediated chain elongation to produce MCCAs was demonstrated here using expired dairy and beverage waste. The fermentation reactor operated at 43 °C was highly enriched with lactate-producing microbes, mainly *Lactobacillus* and *Lacticaseibacillus*, with a combined relative abundance of more than 90%, which promoted the high concentration and selectivity of lactate (81–96% based on mM C) in the product spectrum through the positive correlations between different OTUs. Lactate, as the sole mediator and carbon source for chain elongation, was efficient for producing MCCAs with microbial community structure dominated by the chain elongation microbes *Megasphaera* and *Caproiciproducens*. The study provided a proof of concept for the valorization of dairy and beverage waste using a two-stage lactate-mediated chain elongation platform.

To fully explore the potential of a chain elongation platform for resource recovery from expired dairy and beverage waste, further tests using non-autoclaved expired dairy/beverage feedstock are warranted, avoiding the energy-intensive pretreatment processes. Scaling-up efforts are required after further optimization of the biosystem operation and MCCA extraction, which are critical to reducing capital and operating expenditures. At the same time, a better understating of the mechanism of chain elongation and the discovery of more efficient chain-elongating microbes would be beneficial to develop better strategies for enhancing MCCA production rates and open the opportunity for the sustainable production of value-added biochemicals and biofuels from high-strength organic wastes.

## CRediT authorship contribution statement

**Bin Bian:** Conceptualization, Investigation, Visualization, Writing - Original Draft. **Wenxiang Zhang:** Investigation. **Najiaowa Yu:** Investigation. **Wei Yang:** Investigation. **Jiajie Xu:** Investigation. **Bruce E. Logan:** Writing - Review & Editing. **Pascal E. Saikaly:** Supervision, Writing - Review & Editing.

## Declaration of competing interest

The authors declare that they have no known competing financial interests or personal relationships that could have appeared to influence the work reported in this paper.

## Appendix A Supplementary data

Bian et al_SI_ESE-D-23-00793.pdf Bian et al_SI_ESE-D-23-00793.pdf
