# Supplementary material for: Lactate-mediated medium-chain fatty acid production from expired dairy and beverage waste
Source: Environ Sci Ecotechnol. 2024 Apr 23;21:100424. doi: 10.1016/j.ese.2024.100424 (PMC11106833; doi:10.1016/j.ese.2024.100424)
Supplement: Bian et al_SI_ESE-D-23-00793.pdf [file mmc1.pdf]

Lactate-mediated medium-chain fatty acid production from expired dairy and beverage waste

Bin Bian[a, c#,*], Wenxiang Zhang[a, d#], Najiaowa Yu[c], Wei Yang[a], Jiajie Xu[a, e], Bruce E. Logan[c], Pascal E. Saikaly[a, b*]

[a] *Water Desalination and Reuse Center (WDRC), King Abdullah University of Science and Technology (KAUST), Thuwal 23955-6900, Kingdom of Saudi Arabia*

[b] *Environmental Science and Engineering Program, Biological and Environmental Science and Engineering (BESE) Division, King Abdullah University of Science and Technology (KAUST), Thuwal 23955-6900, Kingdom of Saudi Arabia*

[c] *Department of Civil and Environmental Engineering, The Pennsylvania State University, University Park, PA, 16802, USA*

[d] *Research Centre of Ecology & Environment for Coastal Area and Deep Sea, Southern Marine Science and Engineering Guangdong Laboratory (Guangzhou), Guangzhou, 511458, China*

[e] *School of Marine Science, Ningbo University, Ningbo, 315211, China*

[#] *These authors contributed equally to this work.*

[*] *Corresponding author: Pascal E. Saikaly. E-mail: pascal.saikaly@kaust.edu.sa; Bin Bian. bin.bian@kaust.edu.sa.*

1. Reactor construction and operation

1.1 Continuous operation of fermentation reactor

For the continuous operation of the fermentation reactor, first, three different temperatures were tested for their impact on lactate concentration and production rate using a heating jacket: Period F-I (30 ˚C), Period F-II (35 ˚C), and Period F-III (43 ˚C). The CM medium stored at 4 ˚C was diluted with tap water (TW) at a ratio of 1:2 before being used as the influent to the fermentation reactor in Period F-I to F-III, and the hydraulic retention time (HRT) was 4 days. Next, we examined the effect of increasing the organic loading rate on the performance of the fermentation reactor. The HRT was adjusted to 2 days in Period F-IV and F-V, and to further increase the organic loading rate CM with no dilution was used as a feedstock in Period F-V.

1.2 Feed substrates during Periods CE-I to CE-VI in chain elongation reactor

Synthetic medium containing 800 mM-C ethanol (EtOH, Sigma-Aldrich) and 400 mM-C sodium acetate (Ac, Sigma-Aldrich) was initially fed into the reactor in Period CE-I to enrich for chain elongation microbes. Given that the main product in the fermentation reactor was lactate, the influent for the chain elongation reactor was adjusted in Periods CE-II to CE-V by gradually adding DL-lactic acid (LA, Sigma-Aldrich) to 1050 mM-C and reducing the concentration of ethanol and acetate to 0 mM-C in Period CE-V. In Period CE-VI, unfiltered and unsterilized fermentation reactor effluent, containing around 810-840 mM-C lactate, was used as the feed for the chain elongation reactor, to test the feasibility of chain elongation platform for the production of MCCAs through a two-stage fermentation process using expired dairy and beverage waste as raw materials.

1.3 MCCA extraction from chain elongation reactor

One external extraction system made of hollow-fiber membranes was constructed for the continuous forward and reverse extraction of MCCAs from the chain elongation bioreactor, similar to our previous report [1]. The chain elongation broth was continuously circulated through the exterior space of the forward membrane module at a flow rate of 50 ml min^{-1}. Mineral oil solvent (VWR) with 3% tri-n-octylphosphine oxide (TOPO) (Alfa Aesar) was used as the hydrophobic forward extraction solvent at an upflow rate of 30 ml min^{-1}, while the alkaline solution buffered

with 0.2 M boric acid was utilized for the reverse extraction, which was maintained at a pH of 9-11 with manual addition of 2 M sodium hydroxide solution every 4-7 days.

2. DNA extraction and sequencing

2.1 DNA extraction

DNA extraction of the samples was done using a slightly modified version of the standard protocol for FastDNA Spin kit for Soil (MP Biomedicals, USA) with the following exceptions: 500 µL of sample, 480 µL Sodium Phosphate Buffer and 120 µL MT Buffer were added to a Lysing Matrix E tube. Bead beating was performed at 6 m/s for 4x40s [2]. Gel electrophoresis using Tapestation 2200 and Genomic DNA screentapes (Agilent, USA) was used to validate product size and purity of a subset of DNA extracts. DNA concentration was measured using Qubit dsDNA HS/BR Assay kit (Thermo Fisher Scientific, USA).

2.2 Sequencing library preparation

Amplicon libraries for the archaea/bacteria/eukarya 16S/18S rRNA gene variable regions 4-8 (abeV48-A) were prepared using a custom protocol. Up to 25 ng of extracted DNA was used as template for PCR amplification of the archaea/bacteria/eukarya 16S/18S rRNA gene variable regions 4-8 (abeV48-A). Each PCR reaction (50 µL) contained 0.5 mM dNTP mix, 0.01 units of Platinum SuperFi DNA Polymerase (Thermo Fisher Scientific, USA), and 500 nM of each forward and reverse primer in the supplied SuperFI Buffer. PCR was done with the following program: Initial denaturation at 98 °C for 3 min, 25 cycles of amplification (98 °C for 30 s, 62 °C for 20 s, 72 °C for 2 min) and a final elongation at 72 °C for 5 min. The forward and reverse primers used include custom 24 nt barcode sequences followed by the sequences targeting the archaea/bacteria/eukarya 16S/18S rRNA gene variable regions 4-8 (abeV48-A): [515FB] GTGYCAGCMGCCGCGGTAA and [1391R] GACGGGCGGTGWGTRCA [3, 4]. The resulting amplicon libraries were purified using the standard protocol for CleanNGS SPRI beads (CleanNA, NL) with a bead to sample ratio of 3:5. DNA was eluted in 25 µL of nuclease free water (Qiagen, Germany). Sequencing libraries were prepared from the purified amplicon libraries using the SQK-LSK110 kit (Oxford Nanopore Technologies, UK) according to manufacturer protocol with the following modifications: 500 ng total DNA was used as input, and CleanNGS SPRI beads for library clean-up steps. DNA concentration was measured using Qubit dsDNA HS Assay kit

(Thermo Fisher Scientific, USA). Gel electrophoresis using Tapestation 2200 and D1000/High sensitivity D1000 screentapes (Agilent, USA) was used to validate product size and purity of a subset of amplicon libraries.

2.3 DNA sequencing

Circa 12 ng (~20 fmol) of the resulting sequencing library was loaded onto a MinION R10.4.1 flowcell and sequenced using the MinKNOW v22.03.6 software (Oxford Nanopore Technologies, UK). Reads were base-called and demultiplexed with MinKNOW guppy v. 6.0.7 using the super accurate basecalling algorithm (configr10.4.1_450bps_sup.cfg) and custom barcodes.

2.4 Bioinformatic processing of sequence reads

Sequencing reads in the demultiplexed and basecalled fastq files were length filter for length (320 - 2000 bp) and quality (phred score > 15) using a local implementation of filtlong v0.2.1 with the settings **–min_length 700 –max_length 2000 –min_mean_q 97**. The filtered reads were mapped to the QIIME-formatted MiDAS database, release 4.8.1 [5-8] with minimap2 v2.24-r1122 using the **-ax map-ont** command [9] and downstream processing using samtools v1.14 [10]. Mapping results were filtered such that query sequence length relative to alignment length deviated < 5 %. Noteworthy, low abundant operational taxonomic units (OTUs) making up < 0.01 % of the total mapped reads within each sample were disregarded as a data denoising step. Further bioinformatic processing was done via RStudio IDE (2022.2.3.492) running R version 4.2.2 (2022-10-31) and using the R packages: ampvis2 (2.7.27) [1], tidyverse (1.3.1), seqinr (4.2.16), ShortRead (1.54.0) and iNEXT (2.0.20) [11, 12].

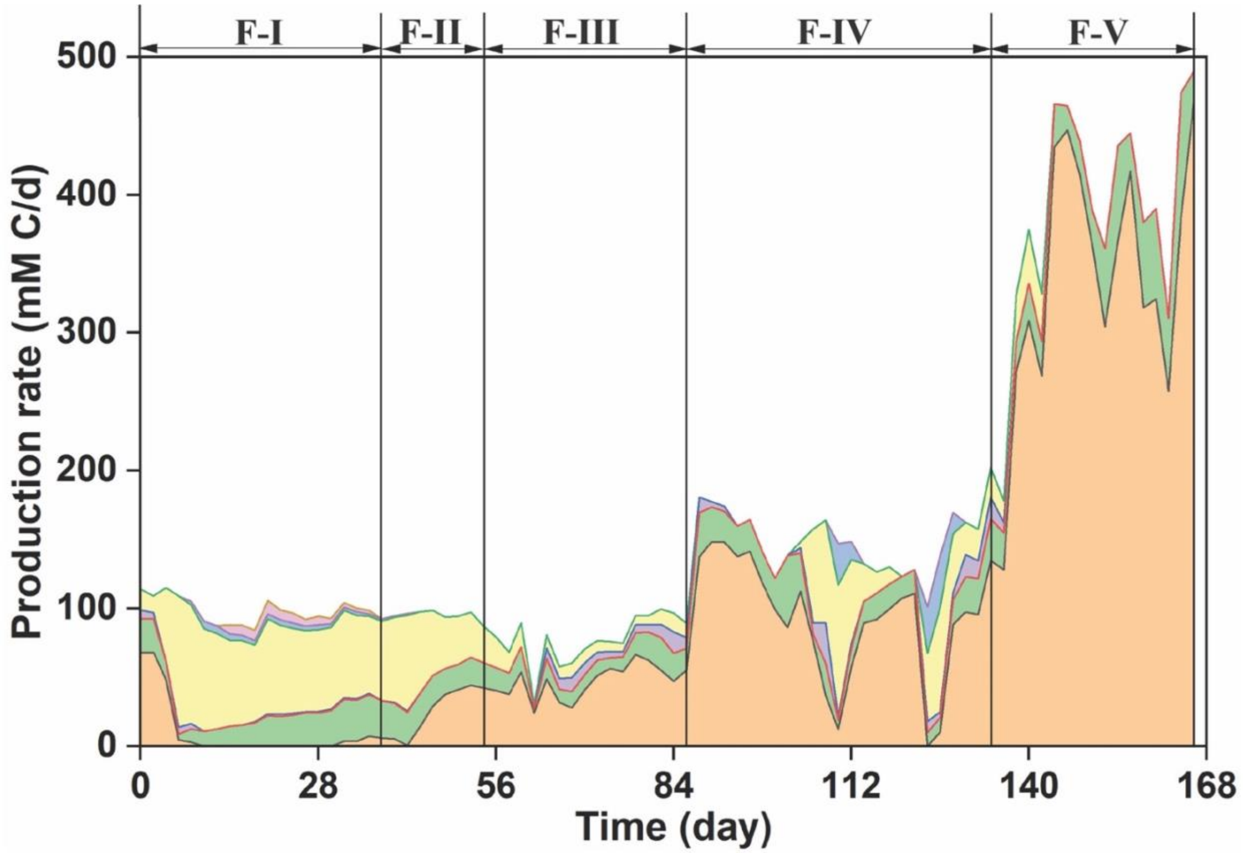

Figure S1 Stacked area charts for the production rate of lactate and volatile fatty acids during Period F-I to F-V in the fermentation reactor.

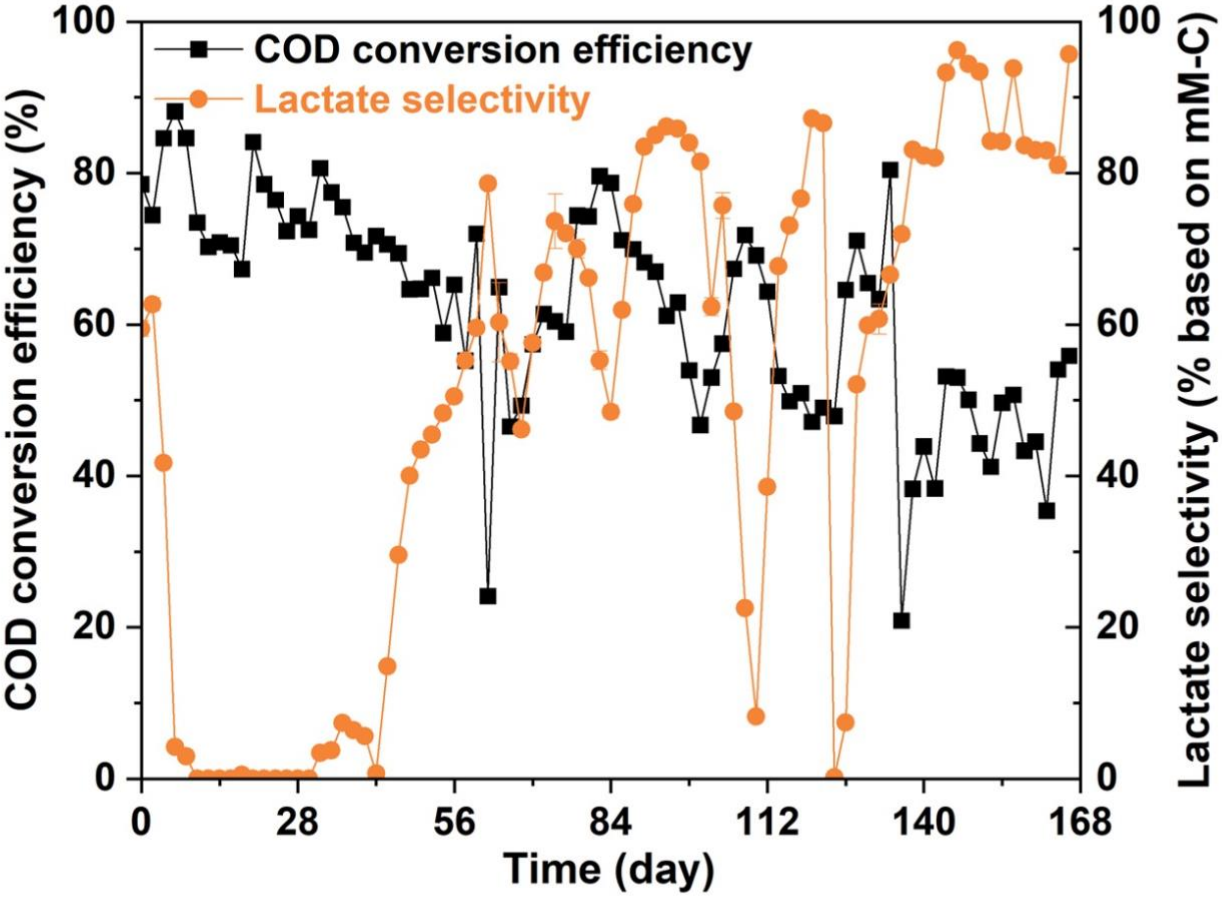

Figure S2 COD conversion efficiency and lactate selectivity calculated based on mM-C among all VFAs and lactate during Period F-I to F-V in the fermentation reactor.

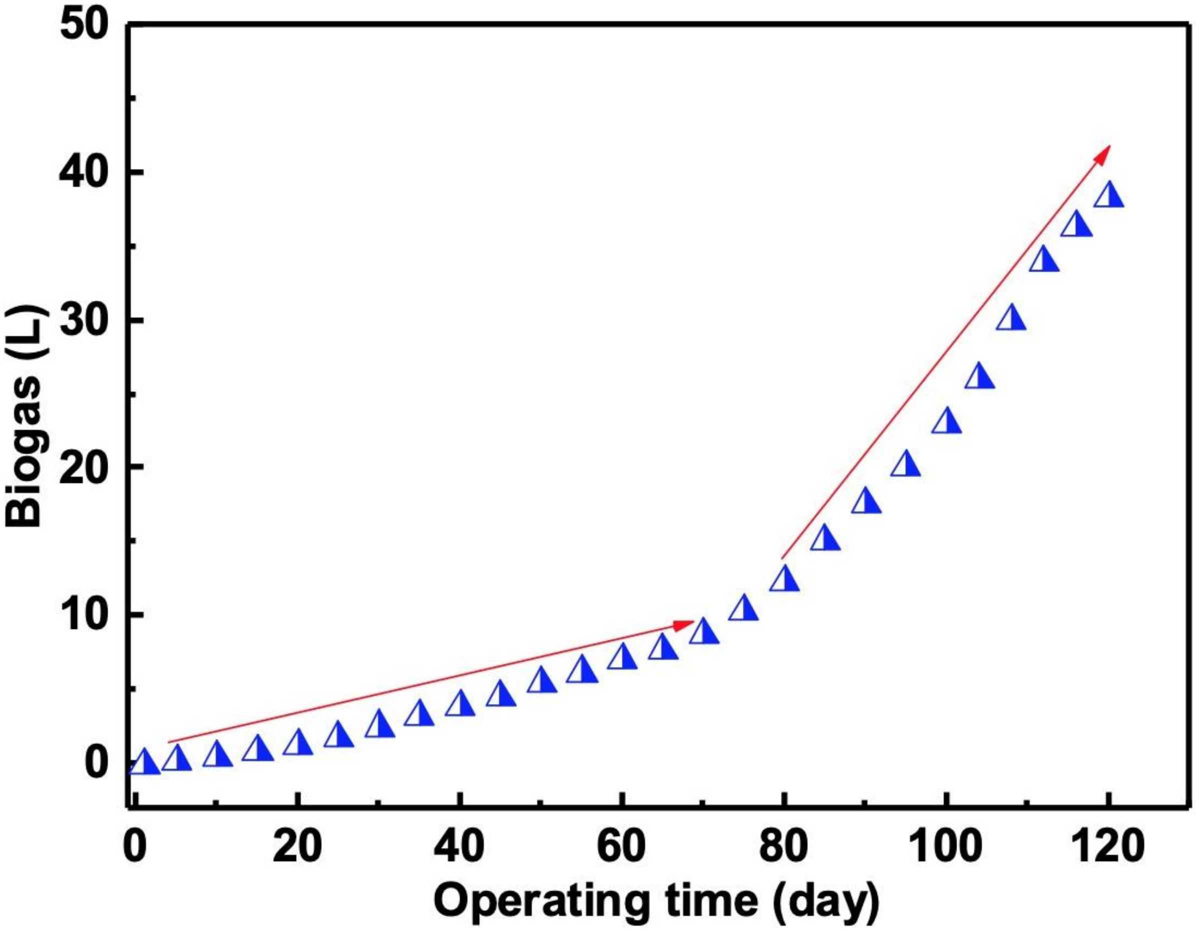

Figure S3 Biogas production from chain elongation reactor. Gas composition: H_2: ~81%, N_2: ~3%, and CH_4: 16%.

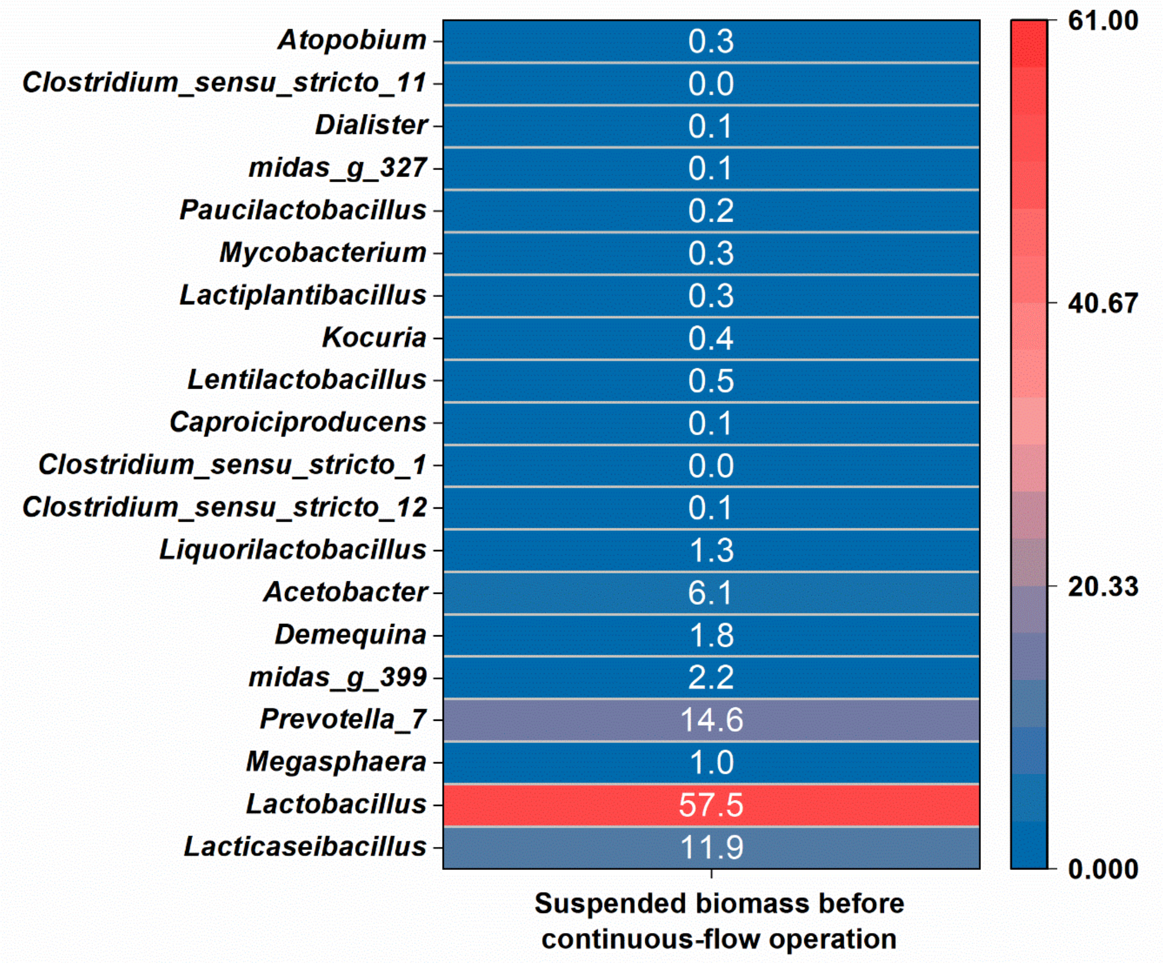

Figure S4 Heatmap of the relative abundance of the top 20 operational taxonomic units in the suspended biomass of the fermentation reactor before switching operation to continuous mode.

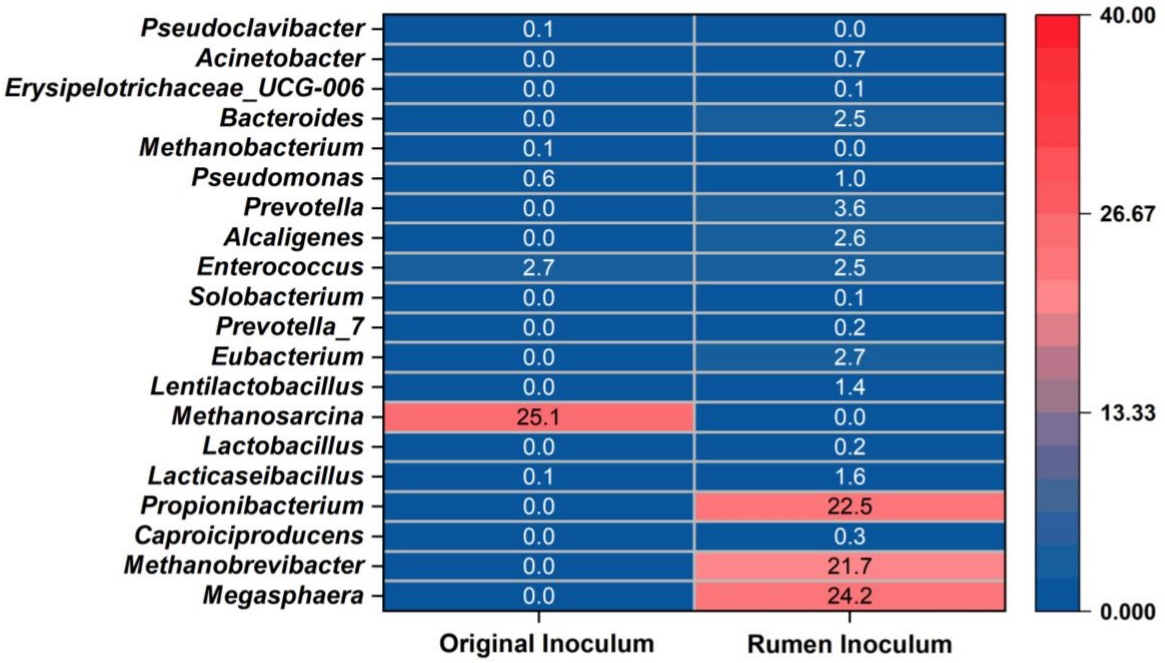

Figure S5 Heatmap of the relative abundance of the top 20 operational taxonomic units in the inoculums used to seed the chain elongation reactor.

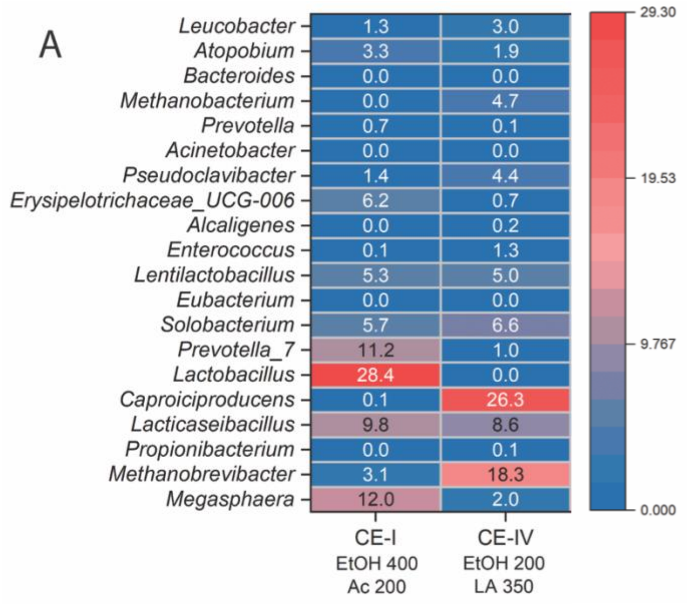

Figure S6 Heatmap of the relative abundance of the top 20 operational taxonomic units in the suspension of the chain elongation reactor during Period CE-I and CE-IV. The taxa level shown on the left-hand side represents the genus level.

Table S1 Comparison of caproate production via lactate-driven chain elongation

Substrate	Reactor type	No. of days	pH	Dominant microbiome	RA[d] (%)	Max concentration (g L^{-1})	Max production (g L^{-1} day^{-1})	Ref.
Thin stillage + H_2	1 L reactor (with electro chemical cells)	42	5.4~5.7	*Megasphaera elsdenii*	57	2.08	0.58	[13]
L-Lactate	0.7 L anaerobic filter	220	5.0	*Acinetobacter* sp.	62.9	N/A[c]	3.03	[14]
Grass	100 ml reactor	30	5.5~6.2	*Clostridium* IV	28	4.09	N/A	[15]
Acid whey	0.7 L CSTR[a]	90	5.0	*Bacteroidales*	21.7	N/A	1.68	[16]
15× diluted food waste leachate + H_2	6 L leach-bed	100	Initial pH 7.0	*Clostridiales*<br>*Clostridium*	12.6<br>42	9.59	N/A	[17]
Acid whey	1 L UASB[b]	400	5.5	*Ruminococcaceae*<br>*Prevotellaceae*	20.5<br>17.9	10.4	3.20	[18]
Liquor brewing wastewater	5 L anaerobic filter	485	6.5	*Clostridium* IV	25.5	3.59	10.9	[19]
Xylan + lactate	1 L CSTR	148	5.5	*Ruminiclostridium 5*	42.3	N/A	3.60	[20]
10–15% (vol/vol) food waste	1 L stirred tank reactor	45	6.0	*Caproiciproducens*	32–72	5.4	1.81	[21]
Lactate	0.9 L CSTR	194	5.0–6.5	*Caproiciproducens*	2–67.8	8.58	N/A	[22]

Substrate	Reactor type	No. of days	pH	Dominant microbiome	RA[d] (%)	Max concentration (g L^{-1})	Max production (g L^{-1} day^{-1})	Ref.
DL-Lactate	3.0-liter CSTR	780	6.0	*Caproiciproducens*	53.9	33.7	11.5	[23]
Liquor brewing wastewater	2.5 L anaerobic filter	377	6.0	*Caproiciproducens*	60.9	19.7	25.4	[24]
	500 L fermentation reactor	24		N/A	N/A	14.9	N/A	
Expired dairy/ beverage waste	10 L UASB	105	5.5-6	*Megasphaera*	2–29.3	14.8	5.9	This study

[a] CSTR, continuously stirred tank reactor. [b] UASB, upflow anaerobic sludge blanket. [c] N/A, data not available. [d] RA, relative abundance of the dominant caproate-producing microbial species.

References

[1] J. J. Xu, B. Bian, L. T. Angenent and P. E. Saikaly. Long-term continuous extraction of medium-chain carboxylates by pertraction with submerged hollow-fiber membranes. Front. Bioeng. Biotechnol., 9(716) (2021) 726946.

[2] M. Albertsen, S. M. Karst, A. S. Ziegler, R. H. Kirkegaard and P. H. Nielsen. Back to Basics – The Influence of DNA Extraction and Primer Choice on Phylogenetic Analysis of Activated Sludge Communities. PLoS One, 10(7) (2015) e0132783.

[3] A. Apprill, S. McNally, R. Parsons and L. Weber. Minor revision to V4 region SSU rRNA 806R gene primer greatly increases detection of SAR11 bacterioplankton. Aquat. Microb. Ecol., 75(2) (2015) 129-137.

[4] A. E. Parada, D. M. Needham and J. A. Fuhrman. Every base matters: assessing small subunit rRNA primers for marine microbiomes with mock communities, time series and global field samples. Environ. Microbiol., 18(5) (2016) 1403-1414.

[5] M. S. Dueholm, K. S. Andersen, S. J. McIlroy, J. M. Kristensen, E. Yashiro, S. M. Karst, M. Albertsen and P. H. Nielsen. Generation of comprehensive ecosystem-specific reference databases with species-level resolution by high-throughput full-length 16S rRNA gene sequencing and automated taxonomy assignment (AutoTax). MBio, 11(5) (2020) e01557-01520.

[6] M. K. D. Dueholm, M. Nierychlo, K. S. Andersen, V. Rudkjøbing, S. Knutsson, M. Albertsen and P. H. Nielsen. MiDAS 4: A global catalogue of full-length 16S rRNA gene sequences and taxonomy for studies of bacterial communities in wastewater treatment plants. Nat. Commun., 13(1) (2022) 1-15.

[7] S. J. McIlroy, R. H. Kirkegaard, B. McIlroy, M. Nierychlo, J. M. Kristensen, S. M. Karst, M. Albertsen and P. H. Nielsen. MiDAS 2.0: an ecosystem-specific taxonomy and online database for the organisms of wastewater treatment systems expanded for anaerobic digester groups. Database, 2017 (2017).

[8] J. G. Caporaso, J. Kuczynski, J. Stombaugh, K. Bittinger, F. D. Bushman, E. K. Costello, N. Fierer, A. G. Peña, J. K. Goodrich and J. I. Gordon. QIIME allows analysis of high-throughput community sequencing data. Nat. Methods, 7(5) (2010) 335-336.

[9] H. Li. Minimap2: pairwise alignment for nucleotide sequences. Bioinformatics, 34(18) (2018) 3094-3100.

[10] P. Danecek, J. K. Bonfield, J. Liddle, J. Marshall, V. Ohan, M. O. Pollard, A. Whitwham, T. Keane, S. A. McCarthy and R. M. Davies. Twelve years of SAMtools and BCFtools. Gigascience, 10(2) (2021) giab008.

[11] T. Hsieh, K. Ma and A. Chao. iNEXT: an R package for rarefaction and extrapolation of species diversity (H ill numbers). Methods Ecol. Evol., 7(12) (2016) 1451-1456.

[12] A. Chao, N. J. Gotelli, T. Hsieh, E. L. Sander, K. Ma, R. K. Colwell and A. M. Ellison. Rarefaction and extrapolation with Hill numbers: a framework for sampling and estimation in species diversity studies. Ecol. Monogr., 84(1) (2014) 45-67.

[13] S. J. Andersen, P. Candry, T. Basadre, W. C. Khor, H. Roume, E. Hernandez-Sanabria, M. Coma and K. Rabaey. Electrolytic extraction drives volatile fatty acid chain elongation through lactic acid and replaces chemical pH control in thin stillage fermentation. Biotechnol. Biofuels, 8(1) (2015) 221.

[14] L. A. Kucek, M. Nguyen and L. T. Angenent. Conversion of L-lactate into n-caproate by a continuously fed reactor microbiome. Water Res., 93 (2016) 163-171.

[15] W. C. Khor, S. Andersen, H. Vervaeren and K. Rabaey. Electricity-assisted production of caproic acid from grass. Biotechnol. Biofuels, 10(1) (2017) 180.

[16] J. J. Xu, J. X. Hao, J. J. L. Guzman, C. M. Spirito, L. A. Harroff and L. T. Angenent. Temperature-phased conversion of acid whey waste into medium-chain carboxylic acids via lactic acid: no external e-donor. Joule, 2(2) (2018) 280-295.
[17] C. O. Nzeteu, A. C. Trego, F. Abram and V. O'Flaherty. Reproducible, high-yielding, biological caproate production from food waste using a single-phase anaerobic reactor system. Biotechnol. Biofuels, 11(1) (2018) 108.
[18] A. Duber, L. Jaroszynski, R. Zagrodnik, J. Chwialkowska, W. Juzwa, S. Ciesielski and P. Oleskowicz-Popiel. Exploiting the real wastewater potential for resource recovery – n-caproate production from acid whey. Green Chem., 20(16) (2018) 3790-3803.
[19] Q. Wu, X. Feng, W. Guo, X. Bao and N. Ren. Long-term medium chain carboxylic acids production from liquor-making wastewater: Parameters optimization and toxicity mitigation. Chem. Eng. J., 388 (2020) 124218.
[20] B. Liu, S. Kleinsteuber, F. Centler, H. Harms and H. Sträuber. Competition between butyrate fermenters and chain-elongating bacteria limits the efficiency of medium-chain carboxylate production. Front. Microbiol., 11 (2020) 336.
[21] C. A. Contreras-Dávila, V. J. Carrión, V. R. Vonk, C. N. J. Buisman and D. P. B. T. B. Strik. Consecutive lactate formation and chain elongation to reduce exogenous chemicals input in repeated-batch food waste fermentation. Water Res., 169 (2020) 115215.
[22] P. Candry, L. Radić, J. Favere, J. M. Carvajal-Arroyo, K. Rabaey and R. Ganigué. Mildly acidic pH selects for chain elongation to caproic acid over alternative pathways during lactic acid fermentation. Water Res., 186 (2020) 116396.
[23] X. Zhu, X. Feng, C. Liang, J. Li, J. Jia, L. Feng, Y. Tao and Y. Chen. Microbial Ecological Mechanism for Long-Term Production of High Concentrations of n-Caproate via Lactate-Driven Chain Elongation. Appl. Environ. Microbiol., 87(11) (2021) e03075-03020.
[24] X. Zhu, H. Huang, Y. He, X. Wang, J. Jia, X. Feng, D. Li and H. Li. A preliminary study on the feasibility of industrialization for n-caproic acid recovery from food wastewater: From lab to pilot. Bioresour. Technol., 366 (2022) 128154.
